# Multi-Cellular Functions of MG53 in Muscle Calcium Signaling and Regeneration

**DOI:** 10.3389/fphys.2020.583393

**Published:** 2020-11-06

**Authors:** Dathe Z. Benissan-Messan, Hua Zhu, Weina Zhong, Tao Tan, Jianjie Ma, Peter H. U. Lee

**Affiliations:** ^1^Department of Surgery, The Ohio State University, Columbus, OH, United States; ^2^Department of Pathology and Laboratory Medicine, Brown University, Providence, RI, United States; ^3^Department of Cardiothoracic Surgery, Southcoast Health, Fall River, MA, United States

**Keywords:** metabolic syndrome, skeletal muscle regeneration, myogenesis, insulin resistance, mitochondria, calcium homeostasis, plasma membrane repair, TRIM72

## Abstract

Since its identification in 2009, multiple studies have indicated the importance of MG53 in muscle physiology. The protein is produced in striated muscles but has physiologic implications reaching beyond the confines of striated muscles. Roles in muscle regeneration, calcium homeostasis, excitation-contraction coupling, myogenesis, and the mitochondria highlight the protein’s wide-reaching impact. Numerous therapeutic applications could potentially emerge from these physiologic roles. This review summarizes the current literature regarding the role of MG53 in the skeletal muscle. Therapeutic applications are discussed.

## Introduction

More than 70 TRIM family proteins exist. They are characterized by the presence of a tripartite motif composed of a RING finger, B-box motifs, and a coiled coil region at their N-terminus. Among them is a 53 kD protein named Mitsugumin 53 (MG53) (Cai et al., 2009a; McNeil, 2009). MG53, also known as TRIM72, was cloned in 2009 using an immunoproteomic library in an attempt to identify proteins involved in myogenesis, calcium signaling, and striated muscle integrity (Weisleder et al., 2008; Tan et al., 2016). Since its identification, multiple physiologic functions have been attributed to this protein, which have made it an attractive candidate in translational medicine as a potential therapeutic agent. The protein is primarily expressed in striated muscles. This review of literature details the current understanding of the role of MG53 in skeletal muscle. Therapeutic applications of the protein relative to skeletal muscle diseases are discussed.

## The Skeletal Muscle

Skeletal muscles are made of bundles of contractile units called myofibers. Myofibers are made of myofibrils which are in turn composed of longitudinal thick and thin filaments (Ciciliot and Schiaffino, 2010). Myofibers form fascicles and bundles of fascicles form muscle tissue (Mukund and Subramaniam, 2020). Myofibers arise during development from the fusion of mononucleate precursor cells known as myoblasts and satellites cells which are stem cells that are activated during regeneration following muscle injury to proliferate and differentiate into myofibers (Blanco-Bose et al., 2001). Skeletal muscles are highly vascularized and innervated (Mukund and Subramaniam, 2020).

Contraction of muscle fibers occur following conduction of impulses through motor neurons via neuromuscular junctions through excitation-contraction (EC) coupling (Takeshima et al., 1994; Mukund and Subramaniam, 2020). Acetylcholine receptors in the plasma membrane of skeletal muscle cells are activated by the release of acetylcholine from motor neurons and leads to membrane depolarization (Lee, 2010; Ahn et al., 2016). The membrane potential induced through depolarization is propagated to the interior of muscle cells via transverse tubules (T-tubules) and leads to the activation of dihydropyridine receptors (DHPR), which in turn activate ryanodine receptor 1 (RyR1), a calcium channel on the sarcoplasmic reticulum membrane (Ahn et al., 2016). RyR1 is responsible for the release of calcium ions into the cytosol (Takeshima et al., 1994). Calcium ions in the cytosol further activate more RyR1 to release more calcium into the cytosol, a phenomenon referred to as calcium-ion induced calcium release (Takeshima et al., 1994). Entry of extracellular calcium into the cell through store-operated calcium entry (SOCE) via Orai1 or through the transient receptor potential canonical (TRPC) channels also contribute to the calcium supply during skeletal muscle contraction (Ahn et al., 2016). Following contraction, the sarcoplasmic endoplasmic reticulum calcium ion ATPase 1a (SERCA1a) uptakes cytosolic calcium and leads to a return to a resting level and a replenishment of the calcium content of the sarcoplasmic reticulum (Ahn et al., 2016).

In addition to being a contractile machine, the skeletal muscle is considered an endocrine organ and secretes several myokines that modulate tissue function (Iizuka et al., 2014; Karstoft and Pedersen, 2016; Giudice and Taylor, 2017; Hoffmann and Weigert, 2017; Bian et al., 2019). Striated muscles are constantly subjected to stress and an active injury-repair mechanism is necessary for their optimum function. Repeated use, stress, diseases, and other injuries would otherwise impair muscle function and movement. The repair of the plasma membrane following injury requires the translocation of intracellular vesicles to the site of injury (Cai et al., 2009a,b). These vesicles fuse with the plasma membrane in a calcium dependent manner (Cai et al., 2009a). Dysferlin and caveolae are essential components of the membrane repair machinery and participate in vesicle fusion to the plasma membrane (Bansal et al., 2003; Glover and Brown, 2007).

## MG53 in the Skeletal Muscle

MG53 is composed of a TRIM domain at the N-terminus, a PRY domain (associated with SPRY), and a SPRY domain (sequence repeat in the dual-specificity kinase sp1A and ryanodine receptor) at the C-terminus (Cai et al., 2009a; Ahn et al., 2016). Generally, TRIM containing proteins control important cellular processes in intracellular signaling, innate immunity, transcription, autophagy, and carcinogenesis (Hatakeyama, 2017). Genetic analysis of the TRIM domain reveals that it is made up of a Really Interesting New Gene (RING) domain with ubiquitin E3 ligase activity, a B-box with a zinc-binding domain, and coiled-coil moieties (Ozato et al., 2008; Cai et al., 2009a; Tan et al., 2016). The RING finger domain is a zinc finger motif containing a Cys_3_HisCys_4_ amino acid motif which binds two zinc cations (Zhang et al., 2017). The E3 ubiquitin ligase activity of the RING domain catalyzes ubiquitin conjugation which targets proteins for degradation (Hatakeyama, 2017). The B-box domains are zinc binding as well and have been shown to play roles in the innate immune system defense (Ozato et al., 2008). The coiled-coil domain mediates interactions among TRIM proteins which leads to protein assembly into high molecular mass complexes (Ozato et al., 2008; Zhang et al., 2017). The PRY domain is involved in protein binding and has been suggested to be an interface for the protein-protein interaction of MG53 with other proteins (Ahn et al., 2016). At the C-terminus, MG53 has a SPRY domain, which is responsible for the binding of target proteins (Zhang et al., 2017). MG53 is primarily produced in cardiac and skeletal muscles (Cai et al., 2009a; Hu and Xiao, 2018). RNA analysis has revealed expression in the lung and kidney (Cai et al., 2009a; Jia et al., 2014; Duann et al., 2015). The protein was also noted to be expressed by macrophages (Sermersheim et al., 2020). Recombinant human MG53 (rhMG53) has been purified and is available for therapeutic applications (Weisleder et al., 2012; Jia et al., 2014; Duann et al., 2015; Li et al., 2015; Liu J. et al., 2015; Zhu et al., 2015; Yao et al., 2016; Adesanya et al., 2019; Bian et al., 2019; Chandler et al., 2019; Guan et al., 2019a,b; Liu et al., 2020; Sermersheim et al., 2020).

### The Role of MG53 in Skeletal Muscle Calcium Homeostasis and EC Coupling

**FIGURE 1:**
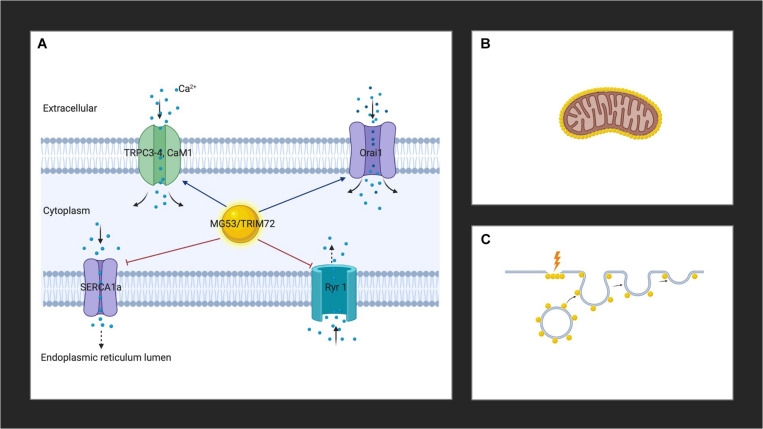
**(A)** MG53 controls cytosolic calcium through interactions with Orai1, RyR1, TRPC3, TRPC4, CaM1, and SERCA. **(B)** MG53 aggregates around the mitochondria of skeletal muscle in the presence of reactive oxygen species. **(C)** MG53 promotes plasma membrane repair by facilitating vesicle translocation following injury. Golden spheres represent MG53. Created with BioRender.com.

MG53 binds to Orai1 via its PRY-SPRY region and co-localizes with Orai1 to the plasma membranes of skeletal muscles to enhance extracellular calcium entry via by SOCE, while reducing intracellular calcium release through RyR1, and increasing the expression of TRPC3, TRPC4, and calmodulin 1(CaM1) (Figure 1A; Ahn et al., 2016). MG53 also attenuates SERCA1a activity, leading to a rise in cytosolic calcium (Lee et al., 2012). The TRIM and PRY domains of MG53 constitute the binding region to SERCA1a and the interaction of MG53 and SERCA1a reduces the activity of SERCA1a, which takes up calcium from the cytosol to the sarcoplasmic reticulum (Lee et al., 2012; Ahn et al., 2016). Together, this leads to a transient increase in cytosolic calcium, which results in efficient skeletal muscle contractions (Ahn et al., 2016).

### The Role of MG53 in the Mitochondria

Studies in high fat diet (HFD)-induced metabolic syndrome mouse models have demonstrated an aggregation of MG53 around the mitochondria of skeletal muscle in the presence of reactive oxygen species (ROS) which suggests a role for the protein as a mitochondrial guardian (Figure 1B; Ma et al., 2015). Since HFD feeding induces an elevation in mitochondrial activity as a result of increased production of ROS, MG53 is thought to play a protective role (Ma et al., 2015). More recently, MG53 overexpression was also suggested to promote mitochondrial autophagy to help remove damaged mitochondria to relieve CKD-induced muscle atrophy in skeletal muscle cells by upregulating autophagy and beclin 1 regulator 1 (Ambra1), a key regulator involved in autophagosome formation (Lijie et al., 2019). These studies herald the opening of new avenues regarding the widening reach of the activity of MG53. Further studies will undoubtedly elucidate the role of the protein in the mitochondria.

### The Role of MG53 in Plasma Membrane Repair

The role of MG53 in the plasma membrane was one of the first discovered physiological functions of the protein. It is now well established that MG53 facilitates vesicle translocation to the plasma membrane where it leads to repair following injury (Figure 1C; Cai et al., 2009a). Cholesterol exposure in the plasma membrane following skeletal muscle injury is thought to mediate the translocation of MG53 upon membrane damage (Han, 2011; Zhu et al., 2011). The protein appears as a monomer in the reduced environment of the cytoplasm of an intact cell, and acute muscle plasma membrane disruption leads to exposure of the cell interior to the external oxidized environment (Cai et al., 2009a). A change in the oxidation state of MG53 ensues, which initiates oligomerization (Cai et al., 2009a; McNeil, 2009). Targeted mutagenesis revealed that MG53 oligomer formation requires a conserved cystidine residue (C242) and mutations in C242 do not result in the formation of oligomers (Matsuda et al., 2012). Oligomerization of MG53 through oxidation of the thiol group of cysteine at 242 and a leucine zipper motif between the two coiled-coil domains induces the nucleation of intracellular vesicles coated with MG53, the trafficking of vesicles to the injury sites, and the resealing of the injured membranes (Hwang et al., 2011; Ahn et al., 2016). At the membrane, MG53 concentrates at the site of injury in damaged adult muscle fibers (Cai et al., 2009a; Weisleder et al., 2012; Lek et al., 2013). The protein binds to phosphatidylserine in the plasma membrane to mediate vesicle accumulation at the site of injury (Cai et al., 2009a; Tan et al., 2016). Polymerase I and transcript release factor (PTRF; also known as cavin-1) recognize cholesterol at the injury site and act as a docking protein to tether MG53 together with its associated vesicles to the damage site (Zhu et al., 2011). Once associated with intracellular vesicles and the inner leaflet of the sarcolemma of striated muscle, fusion is mediated through membrane proteins such as caveolin-3 (Cav-3) (Weisleder et al., 2009). While the exact interaction of MG53 with Cav-3 to control intracellular vesicle trafficking and membrane remodeling in muscle is unknown, the binding of Cav-3 to MG53 is known to be essential for vesicle fusion (Cai et al., 2009b). Together with dysferlin, PTRF, and non-muscle myosin type IIA, MG53 constitute the membrane repair system (Cai et al., 2009c; Zhu et al., 2011; Lin et al., 2012). The ensuing vesicle fusion to the plasma membrane requires the presence of calcium and results in the formation of a membrane patch which seals the site of injury (Cai et al., 2009b; Weisleder et al., 2009; Lek et al., 2013). A recently proposed novel model of membrane repair including MG53 involves the formation of cap and shoulder proteins (Demonbreun and McNally, 2016; Demonbreun et al., 2016). Following membrane disruption, annexins proteins (A1, A2, A5, and A6) bind phospholipids in the presence of calcium ions to form a “cap” at the site of damage (Demonbreun et al., 2016). Repair “shoulder” proteins, such as dysferlin, Epsin 15 homology domain-containing proteins (EHD1, EHD2), MG53, and BIN1 then localizes adjacent to the repair cap to form a membrane repair complex system and seals the injured site (Demonbreun et al., 2016).

### The Role of MG53 in Myogenesis

Mammalian skeletal muscles are made of multinucleated myofibers formed during development by fusion of mononucleated muscle progenitors (Ciciliot and Schiaffino, 2010). Myogenic differentiation is a tightly regulated process in which mononucleated myoblasts expressing myogenic marker proteins [MyoD, Myogenin, Myosin Heavy Chain (MyHC), and others] proliferate and fuse to form multinucleated myotubes. Matured myotubes convert into contractile myofibers (Okino et al., 2020). The formation of new myofibers or myofiber segments following necrosis is called muscle regeneration (Ciciliot and Schiaffino, 2010). Mature skeletal muscle tissues possess the ability to regenerate from the interaction of satellite cells and their surrounding microenvironment (Mukund and Subramaniam, 2020). Satellite cells are characterized by the expression of transcription factors PAX3 and PAX7 and are located between the basal lamina and the sarcolemma (Relaix et al., 2006; Buckingham, 2007; von Maltzahn et al., 2013). Muscle regeneration following injury is characterized by an inflammatory reaction dominated by the invasion of macrophages, followed by the activation, migration, and proliferation of satellite cells, and their differentiation and fusion into muscle fibers (Ciciliot and Schiaffino, 2010). The maturation of these newly formed myofibers and the remodeling of the regenerated muscle follows (Ciciliot and Schiaffino, 2010). Local apoptosis seen in atrophic muscle can also lead to an increase in the number of satellite cells (Ciciliot and Schiaffino, 2010).

Quiescent satellite cells are marked by the expression of PAX7 as well as several other molecular markers, and is notable for the absence of Myogenic Regulatory Factor (MRF), Myogenic Differentiation1 (MYOD1) and Myogenin (MYOG) (Rocheteau et al., 2015; Mukund and Subramaniam, 2020). Skeletal muscle-specific TGFb family member, myostatin, suppresses satellite cell activation (McCroskery et al., 2003). Sprouty1 (SPRY1), a tyrosine inhibitor kinase, is necessary for maintenance and re-entry of PAX7 satellite cells into quiescence (Shea et al., 2010). Satellite cells are activated in response to injury. Activated satellite cells are characterized by PAX7 and the expression of MRFs [MYOD1, MYOG, and Myogenic Factor (MYF)5] (Mukund and Subramaniam, 2020). PAX7 is absolutely required for normal function of satellite cells in regenerative myogenesis in both neonatal and adult skeletal cells (von Maltzahn et al., 2013). Deficiency in PAX7 results in cell-cycle arrest and precocious differentiation (von Maltzahn et al., 2013). The migration to an injury site and proliferation of satellite cells is governed by chemo-attractants released from the extracellular matrix or inflammatory cells (Mukund and Subramaniam, 2020).

Insulin-like growth factors, IGF-I and IGF-II, are essential for skeletal muscle development, hypertrophy, and regeneration. IGF-I plays a key role in the differentiation of satellite cells and the development, hypertrophy, and regeneration of skeletal muscles (Zhang et al., 2017). Likewise, satellite cells lose their muscle differentiation activity when treated with anti-IGF-II antibody or antisense IGF-II, whereas IGF-I overexpression in mice leads to hypertrophy and hyperplasia, leading to increased muscle mass and force generation (Jung and Ko, 2010). IGF-I and IGF-II transduce cellular signaling through the IGF-I receptor (IGFR) that subsequently recruits insulin receptor substrate-1 (IRS-1) (Yi et al., 2013). The recruited IRS-1 can activate the phosphoinositide 3 kinase (PI(3)K)/Akt and Ras/Raf/MEK/ERK pathways. The Ras/Raf/MEK/ERK pathway controls muscle fiber type whereas the PI(3)K/Akt pathway induces muscle differentiation and hypertrophy (Jung and Ko, 2010). The activated Akt targets a mammalian target of rapamycin (mTOR), glycogen synthase kinase 3β (GSK3β), and Forkhead box O (FOXO) (Jung and Ko, 2010). mTOR activity is not involved in muscle differentiation (Jung and Ko, 2010). Phosphorylated GSK3β and FOXO stimulate muscle hypertrophy and suppress muscle atrophy. IRS-1 overexpression inhibits myogenic differentiation through continuous FOXO inhibition and causes repression of MyHC during differentiation (Okino et al., 2020). Additionally, IRS-1 and insulin signaling is reduced during myogenesis, and myoblasts with higher IRS-1 levels are eliminated during differentiation (Bian et al., 2019; Okino et al., 2020).

Intracellularly, evidence suggests that MG53 is a negative regulator of IGF-induced myogenesis (Jung and Ko, 2010; Lee et al., 2010). The MG53 promoter contains E-boxes [which are short DNA motifs within myogenic *cis*-regulatory element (CREs)] and a Myocyte Enhancer Factor (MEF)-binding site for the myogenic transcription factors MyoD and MEF (Jung and Ko, 2010; Soleimani et al., 2018). MG53 is gradually expressed during myogenesis because the transcription of MG53 requires MyoD and Akt (Jung and Ko, 2010; Hong et al., 2016). MG53 then acts as an E3 ligase, targeting the insulin receptor and IRS-1 for ubiquitin-dependent degradation (Song et al., 2013; Yi et al., 2013). This negatively regulates skeletal myogenesis by reducing IGF-I-initiated IRS-1 tyrosine phosphorylation and Akt phosphorylation without affecting IGF-I-elicited IGFR tyrosine phosphorylation and ERK1/2 phosphorylation (Yi et al., 2013). Myogenesis and IGF-I-induced IRS-1 tyrosine phosphorylation were prevented in C2C12 myoblasts by MG53 overexpression but enhanced by MG53 knockdown (Jung and Ko, 2010). The RING domain of MG53 with E3 ligase activity is necessary for the inhibition of IGF-I-elictied IRS-1 phosphorylation (Yi et al., 2013). MG53-induced ubiquitination and degradation of another kinase, focal adhesion kinase (FAK), is also thought to inhibit myogenesis (Nguyen et al., 2014). FAK regulates heterochromatin remodeling to modulate myogenin expression during skeletal myogenesis and regulate the expression of genes involved in membrane fusion, including caveolae (Nguyen et al., 2014). Its inhibition by MG53 is also thought to reduce myogenesis (Nguyen et al., 2014).

Meanwhile, exogenous MG53 has been noted to facilitate the differentiation of C2C12 skeletal myoblasts to myotubes by enhancing vesicle trafficking and membrane fusion (Cai et al., 2009a; Bian et al., 2019). A recent study published by Bian et al. provided evidence that increased levels of MG53 in circulation do not negatively impact myogenesis (Bian et al., 2019). Transgenic mice with sustained elevation of MG53 in circulation (tPA-MG53) have a longer lifespan compared to their wild type counterparts due to enhanced repair capacity after injury and enhanced muscle regeneration (Bian et al., 2019). The authors also did not observe any changes in IRS-1 activity in the skeletal muscles as a result of MG53 overexpression. MG53 was noted to restore satellite cell proliferation in MG53 knockout (mg53^–/–^) mice (Bian et al., 2019). This evidence led to the hypothesis that MG53 may improve muscle regeneration by modulating the activity of satellite cells (Bian et al., 2019). This resulting dichotomous functions between the intracellular mechanism of MG53 and the effect of extracellular MG53 application remains to be elucidated.

### MG53 in Skeletal Muscle Disease Related Therapies

MG53 is an endogenous protein and therefore therapies based on its use are less likely to elicit an immune system reaction. The recombinant human form of the protein (rhMG53) is readily available for therapeutic application and studies have shown limited toxicity (Weisleder et al., 2012; Alloush and Weisleder, 2013; Jia et al., 2014; Duann et al., 2015; Liu J. et al., 2015; Tan et al., 2016). Here, we present a few studies highlighting the potential therapeutic applications for MG53.

When applied intravenously, rhMG53 can potentially treat multiple pathologies in rodent and large animal disease models (Weisleder et al., 2012; Jia et al., 2014; Duann et al., 2015; Liu J. et al., 2015; Zhu et al., 2015). The myokine has been shown to play a protective role in skeletal muscles, kidneys, lungs, brains, skin, hearts, and corneas (He et al., 2012; Weisleder et al., 2012; Jia et al., 2014; Duann et al., 2015; Li et al., 2015; Yao et al., 2016; Adesanya et al., 2019; Chandler et al., 2019). Exogenously administered rhMG53 travels in the circulation and concentrates at the healing edge of injury sites and contributes to regeneration following injury (Weisleder et al., 2012; Bian et al., 2019). Muscle cells and tissues treated with rhMG53 show resistance to mechanical, chemical, and ultraviolet damage (Weisleder et al., 2012). Intravenous injection of the protein protects skeletal muscles against acute and chronic insults (Weisleder et al., 2012; Zhu et al., 2015; Bian et al., 2019). Weisleder et al. (2012) noted in mouse models that 300 nM rhMG53 was sufficient to protect muscle cells against mechanical damage and prevent muscle injury.

The importance of the protein in membrane repair has been highlighted by multiple studies. MG53-/- mice with targeted protein deletion develop progressive muscle pathology with age characterized by defects in skeletal muscle contractility after injury and defective membrane repair (Cai et al., 2009a). Morphologically, a pathologic increase in the number of central nuclei, a measure of defective muscle repair, and decreased diameter of muscle fibers, were observed in isolated skeletal muscles of older mg53-/- mice when compared to younger counterparts and wild type littermates (Cai et al., 2009a). Following cardiotoxin-induced muscle injury, genetically enhanced tPA-MG53 mice overexpressing MG53 displayed enhanced regeneration when compared to wild type and mg53-/- mice (Bian et al., 2019). The regenerative ability of MG53 is thought to be mediated via satellite cell proliferation (Bian et al., 2019).

Muscular dystrophies are an inherited group of disorders characterized by progressive muscle weakness and atrophy and linked to a loss of function of dystrophin and its associated proteins (Glover and Brown, 2007). Muscular dystrophies can be caused by genetic mutations in over thirty different genes, many of which encode for proteins essential for the integrity of muscle cell structure and membranes (He et al., 2012). Mutations in the dystrophin gene in Duchenne muscular dystrophy (DMD) and Becker muscular dystrophy (BMD), or sarcoglycan genes in limb-girdle muscular dystrophies (LGMDs) lead to muscle sarcolemma destabilization and fragility (He et al., 2012). DMD is an X-linked inherited muscle wasting disorder with limited treatment options characterized by compromised muscle structure and decreased muscle function (Burkin and Wuebbles, 2012; Weisleder et al., 2012). Dysferlinopathies are a heterogenous group of progressive muscular dystrophies characterized by mutations in the DYSF gene, leading to reduced or null expression of the dysferlin protein and resulting in varied phenotypes (Flix et al., 2013). Dysferlin is a type II muscle surface membrane, expressed in the sarcolemma, in intracellular vesicles, and in T-tubules (Glover and Brown, 2007; Han, 2011; Flix et al., 2013). Although dysferlin is important in membrane repair, dysferlin itself does not participate in the recruitment of intracellular vesicles for membrane repair and dysferlin -/- muscle retains accumulation of vesicles near membrane damage sites. Dysferlin is known to interact with several proteins including AHNAK, MG53, and Cav3 (Flix et al., 2013). MG53 and AHNAK interact directly with dysferlin (Flix et al., 2013). Like dysferlin, many mutations of Cav3 have been linked to muscular dystrophy (Cai et al., 2009c). Additionally, Cav3 has also been shown to be essential for MG53-mediated vesicle translocation (Cai et al., 2009c).

Cav3 binds to MG53, which in turn interacts with dysferlin (Flix et al., 2013). In the absence of dystrophin, there is weakening of the attachment of the muscle cells to the surrounding basal lamina, which results in reduced force transmission, loss of muscle integrity, altered cell signaling, and weakened sarcolemma that is easily damaged during muscle contraction (Burkin and Wuebbles, 2012). MG53 interacts with dysferlin to facilitate vesicle trafficking to sites of membrane damage (Cai et al., 2009c). Systemic delivery and muscle specific overexpression of the human MG53 gene by recombinant adeno-associated virus (AAV) vectors enhanced membrane repair, ameliorated pathology, and improved muscle and heart functions in δ-sarcoglycan (δ-sarcoglycan) deficient TO-s hamsters (He et al., 2012). Overexpression of MG53 increased dysferlin levels and facilitated its trafficking to the muscle membrane through the participation of Cav3 (He et al., 2012). In addition, MG53 protected muscle cells by activating cell survival kinases, such as Akt, extracellular signal-regulated kinases (ERK1/2), and glycogen synthase kinase-3β (GSK-3β), and inhibiting proapoptotic protein like Bax (He et al., 2012). Even though MG53 delivery would not constitute a cure for dysferlonopathies, a palliation of pathology could be obtained through its delivery in some sarcoglyconopathies.

Maintenance of plasma membrane integrity is vital for cell survival. The constant stress imposed on the skeletal muscle system over time requires a regular system of maintenance to ensure that it continuously fulfills its mechanical and endocrine functions. Aging, cachexia, sarcopenia and disease states such as cancers are conditions associated with compromised muscle integrity and muscle atrophy where MG53 could play a therapeutic role. An increase in MG53 expression level and other plasma membrane repair proteins has already been observed in sedentary *ad libitum* aging in animal studies by Hord et al. (2016). The authors postulated that the increased requirement for phospholipid membrane repair in the aging skeletal muscle necessitated the increased presence of these proteins. The study of the function of MG53 in these diseases states and conditions could further shed light on the restoration of muscle integrity and function. Given its protective roles, MG53 is an ideal candidate therapeutic agent for maintaining skeletal muscle health.

### Controversial Role of MG53 in Diabetes

Skeletal muscles play an important role in glucose control. They are responsible for 70–90% of insulin-stimulated glucose metabolism (Song et al., 2013; Tan et al., 2016). Song et al. reported increased MG53 expression in animal models and human patients with diabetes (Song et al., 2013). They also showed that an upregulation of MG53 occurred before the onset of diabetes (Song et al., 2013). They proposed that an upregulation of MG53 causes insulin insensitivity through the degradation of IRS-1. These observations have not been substantiated by other investigators, and many others failed to observe an upregulation of MG53 in diabetic animals and humans (Ma et al., 2013, 2015, 2017; Xu et al., 2013; Yi et al., 2013; Yuan et al., 2013; Zabielski et al., 2016). Rather, serum samples from high fat diet induced diabetic mice (Ma et al., 2015) and db/db mice (Wang et al., 2020) showed reduced levels of MG53. It is also well-known that a loss of IRS-1 does not directly result in diabetes, owing to a robust compensatory mechanism among the different IRS subtypes (Tamemoto et al., 1994; Terauchi et al., 1997; Laustsen et al., 2002). A recent proteomic study specifically examined the interactions of IRS-1 in skeletal muscle from normal individuals, obese insulin-resistant non-diabetic control subjects, and patients with type 2 diabetes before and after insulin infusion, and failed to identify an enhancement of MG53 (Caruso et al., 2014). Thus, the mounting evidence do not support the proposed role of MG53 in the regulation of IRS-1 in diabetes.

Liu F. et al. (2015) proposed a different mechanism of MG53 mediated diabetes in 2015. The researchers generated transgenic mice by using the alpha myosin heavy chain (α-MHC) promoter to drive expression of MG53 and found that overexpression of MG53 was powerful enough to induce whole body insulin resistance and compromised glucose uptake (Liu F. et al., 2015). MG53 was posited to enter the nucleus and serve as a transcription factor to activate the peroxisome proliferation-activated receptor alpha (PPAR-α) which in turn mediated excessive lipid uptake induced cytotoxicity. Our group successfully generated a MG53 transgenic mouse line to achieve sustained elevation of circulating MG53 by fusing a tPA secretory peptide at the N-terminus of the MG53 protein (tPA-MG53) (Yao et al., 2016; Bian et al., 2019). tPA-MG53 mice live a healthy lifespan with enhanced tissue repair and regeneration capacity (Yao et al., 2016; Bian et al., 2019). To test the role of elevated circulating MG53 in the development of diabetes, we crossed tPA-MG53 mice with db/db mice and found that tPA-MG53/db/db mice develop diabetes at a rate similar to its db/db littermates, suggesting overexpression of MG53 has negligible effects on the development of diabetes (Wang et al., 2020). A separate transgenic mouse model with overexpression of MG53 was generated by Ham and Mahoney (2013). While they used the same α-MHC promoter to drive overexpression of MG53 as Liu F. et al. (2015), they observed that IRS-1 protein levels were higher in transgenic animals when compared to that of their wild type littermates (Ham and Mahoney, 2013). This observation suggests that a feedback mechanism compensates for the downregulation of IRS-1 by MG53. Other studies with mg*53-/-*, tPA-MG53, and their wild type littermates also failed to show alteration of PPAR-α (Bian et al., 2019).

Wu et al. (2019) recently implicated MG53 in the development of diabetes. They showed that the binding affinity between MG53 and insulin receptor (IR) is over threefold higher than that of insulin and insulin receptor (Kd: MG53 and IR is 8 nM vs. insulin and IR is 28 nM) (Wu et al., 2019). Thus, MG53 serves as a novel agonist of IR and a competitive antagonist of insulin. If this hypothesis holds true, not only would the authors have successfully identified the receptor for MG53 but they would also have shown that targeting endogenous MG53 could treat diabetes. This would also imply that the high levels of circulating MG53 present in tPA-MG53 mice could block the insulin pathway and greatly facilitate the development of diabetes. However, as mentioned above, tPA-MG53 mice live a healthy lifespan and when crossed with db/db mice, tPA-MG53/db/db mice are indistinguishable from db/db littermates in terms of growth rate, glucose tolerance test, and insulin tolerance test (Bian et al., 2019; Wang et al., 2020). These studies highlight the deep controversies surrounding the protein that will need to be resolved by future studies.

## Conclusion

Much is still unknown regarding the physiologic impact of MG53 in skeletal muscle despite the presence of numerous studies involving the protein. MG53 undoubtedly plays an important role in several arenas of physiology. It is involved in calcium homeostasis, muscle contraction, and regeneration. Its role as an agent in plasma membrane repair is uncontested. Emerging roles in mitochondrial protection further highlight the fact that not all is known about the protein. The therapeutic applications of the protein in treating skeletal muscle pathologies are numerous and include the use of the protein as an agent of repair, in providing a treatment for certain types of dystrophies, and in modulating muscle regeneration.

## Author Contributions

DB-M, JM, and PL designed the manuscript. DB, HZ, WZ, TT, JM, and PL wrote, read, edited, and approved the final version of manuscript. All authors contributed to the article and approved the submitted version.

## Conflict of Interest

JM was the founder of TRIM-edicine, Inc., a university spin-off biotechnology company that is developing recombinant MG53 protein as a therapeutic reagent for regenerative medicine. Patents on the use of MG53 are held by Rutgers University and The Ohio State University. The remaining authors declare that the research was conducted in the absence of any commercial or financial relationships that could be construed as a potential conflict of interest.
